# Role of OmpA2 surface regions of *Porphyromonas gingivalis* in host–pathogen interactions with oral epithelial cells

**DOI:** 10.1002/mbo3.401

**Published:** 2016-09-06

**Authors:** Kathryn L. Naylor, Magdalena Widziolek, Stuart Hunt, Mary Conolly, Matthew Hicks, Prachi Stafford, Jan Potempa, Craig Murdoch, C. W. Ian Douglas, Graham P. Stafford

**Affiliations:** ^1^ School of Clinical DentistryUniversity of SheffieldSheffieldUnited Kingdom; ^2^ Department of MicrobiologyFaculty of Biochemistry, Biophysics and BiotechnologyJagiellonian UniversityKrakowPoland; ^3^ Biomolecular Research CentreSheffield Hallam UniversityCity CampusSheffieldUnited Kingdom; ^4^ Department of Oral Immunology and Infectious DiseasesUniversity of Louisville School of DentistryLouisvilleKentucky

**Keywords:** host–pathogen interaction, OmpA proteins, oral microbiology, periodontal disease, porphyromonas gingivalis

## Abstract

Outer membrane protein A (OmpA) is a key outer membrane protein found in Gram‐negative bacteria that contributes to several crucial processes in bacterial virulence. In *Porphyromonas gingivalis*, OmpA is predicted as a heterotrimer of OmpA1 and OmpA2 subunits encoded by adjacent genes. Here we describe the role of OmpA and its individual subunits in the interaction of *P. gingivalis* with oral cells. Using knockout mutagenesis, we show that OmpA2 plays a significant role in biofilm formation and interaction with human epithelial cells. We used protein structure prediction software to identify extracellular loops of OmpA2, and determined these are involved in interactions with epithelial cells as evidenced by inhibition of adherence and invasion of *P. gingivalis* by synthetic extracellular loop peptides and the ability of the peptides to mediate interaction of latex beads with human cells. In particular, we observe that OmpA2‐loop 4 plays an important role in the interaction with host cells. These data demonstrate for the first time the important role of *P. gingivalis* OmpA2 extracellular loops in interaction with epithelial cells, which may help design novel peptide‐based antimicrobial therapies for periodontal disease.

## 1 Introduction

Periodontal disease is a general term to describe the chronic inflammatory infections of the gingiva, causing destruction of the periodontal tissues and alveolar bone (Williams, 1990) which, if left untreated, can lead to the loss of teeth. More recently, the association between periodontal disease and systemic disease has gained gravity, establishing links between periodontal disease and cardiovascular disease (Li, Kolltveit, Tronstad, & Olsen, 2000), diabetes mellitus (Soskolne & Klinger, 2001) and rheumatoid arthritis (Koziel, Mydel, & Potempa, 2014). Periodontal disease is initiated by the colonization of oral structures, notably the subgingival regions of the oral cavity, by a complex community of bacterial species (Holt & Ebersole, 2005; Socransky, Haffajee, Cugini, Smith, & Kent, 1998). This complex community can undergo a population shift from healthy‐associated to disease‐associated bacteria, known as dysbiosis, that is characterized by the presence of red complex bacteria as detailed by Socransky et al., (1998). (Hajishengallis, Darveau, & Curtis, 2012) Of particular etiological importance to the progression and severity of the disease is the Gram‐negative anaerobe, *Porphyromonas gingivalis;* a member of the red complex bacteria and also considered to be a keystone pathogen in periodontitis (Hajishengallis, 2010; Hajishengallis et al., 2012; Socransky et al., 1998; Yilmaz, 2008). The virulence of *P. gingivalis* is accredited, in part, to the variety of virulence factors associated with the bacterial cell surface, including lipopolysaccharides, proteases such as the gingipains (Chen & Duncan, 2004), major (FimA) and minor (MfaI) fimbriae (Yilmaz, 2003), all of which have been shown to be involved in invasion of host cells (Nakagawa et al., 2002; Njoroge, Genco, Sojar, Hamada, & Genco, 1997); hemagglutinins (Song et al., 2005); and the major outer membrane proteins (Yoshimura, Murakami, Nishikawa, Hasegawa, & Surface, 2009). Several of these cell surface proteins play a significant role in host interaction, but it is the ability of these proteins to instigate adherence and invasion of the host cell that is considered a crucial part of the disease cycle. These proteins exacerbate the development of chronic periodontitis as they are involved in modulating immune responses and by also potentially acting as a reservoir of intracellular bacteria for recolonization of extracellular niches (Huang, Zhang, Dang, & Haake, 2004; Rudney, Chen, & Sedgewick, 2005; Tribble & Lamont, 2010).

In Gram‐negative bacteria several of the surface exposed proteins that are embedded in the outer membrane are composed of domains that form cylindrical beta‐barrel structures (Koebnik, Locher, & Gelder, 2000). Of these outer membrane proteins, one of the most prominent and abundant are the Outer membrane protein A (OmpA) family proteins (Smith, Mahon, Lambert, & Fagan, 2007). OmpA is a major cell surface protein found in a variety of Gram‐negative bacteria and exhibits a number of functions in a range of pathogens, such as influencing biofilm formation (Orme, Douglas, Rimmer, & Webb, 2006) and host–cell interactions in meningitis‐causing *Escherichia coli* K1‐type strains (Prasadarao et al., 1996), binding to host epithelial cells in *Neisseria gonorrhoeae* (Serino et al., 2007), and more broadly in interactions with insect cells by the *E. coli*‐related *Sodalis* insect symbiont (Weiss, Wu, Schwank, Tolwinski, & Aksoy, 2008). An OmpA protein has been identified in *P. gingivalis* as a heterotrimeric protein of two subunits, referred to in this manuscript as OmpA1 and ‐A2 (but originally termed Pgm6/7 or Omp40/41 by others) (Nagano et al., 2005; Veith, Talbo, Slakeski, & Reynolds, 2001) and demonstrates a high degree of structural homology to *Escherichia coli* OmpA (Nagano et al., 2005). Previous studies of *P. gingivalis* OmpA protein have shown its importance in the stability of the bacterial cell membrane (Iwami, Murakami, Nagano, Nakamura, & Yoshimura, 2007), in adherence to the host with a loss of adherence to endothelial cells in an ∆*ompA1A2* mutant (Komatsu et al., 2012) and in our previous study, indicated the potential involvement of OmpA in *P. gingivalis* interactions with human epithelial cells due to the upregulation of *ompA1* and *ompA2* genes in a hyperinvasive subpopulation of *P. gingivalis* (Suwannakul, Stafford, Whawell, & Douglas, 2010). In this study, we present evidence for the first time that *P. gingivalis* OmpA proteins are key in biofilm formation and are important mediators of host–pathogen interactions with human oral epithelial cells in vitro and systemic virulence in vivo. In particular, we demonstrate a significant role for the extracellular loops of the OmpA2 subunit in interaction with host cells.

## 2 Experimental Procedures

### 2.1 Bacterial strains, mammalian cell culture, and growth conditions

*P. gingivalis* ATCC 33277 wild‐type and isogenic mutant strains were grown at 37°C under anaerobic conditions (10% CO_2_, 10% H_2_, 80% N_2_) on blood agar (BA) plates, derived from fastidious anaerobic agar (Lab M) supplemented with 4.5% oxalated horse blood or in brain heart infusion broth supplemented with 0.5% yeast extract, cysteine (250 μg ml^−1^), menadione (1 mg ml^−1^), hemin (1 mg ml^−1^), and erythromycin (10 μg ml^−1^) where appropriate. The immortalized oral epithelial cell line, OK‐F6 (Dickson et al., 2000) was obtained from James G. Rheinwald (Harvard Institute of Medicine, Boston, MA), and cultured in defined keratinocyte serum‐free media (DKSFM) supplemented with DKSFM growth supplement (Corning) and maintained in a humidified atmosphere of 5% CO_2_ at 37°C.

### 2.2 Construction of *P. gingivalis ∆ompA* mutants

Isogenic mutants of *P. gingivalis* were generated, using a DNA construct obtained either through overlap extension PCR or synthesized commercially through gene synthesis (GeneArt^®^ Strings; ThermoFisher Scientific). Overlap extension PCR products were created through PCR amplification of ~500 bp genomic fragments upstream and downstream of the gene to be deleted and fused to the *ermF* marker through PCR, as previously detailed by (Kuwayama et al., 2002) and using primers described in Table 1 where the first codon of *ermF* replaces the native codon, thus ensuring expression of the antibiotic cassette and reducing chances of any polar effects on downstream gene expression. DNA constructs that were synthesised were designed in the same fashion, with the *ermF* marker flanked by the 500 bp upstream and downstream regions. Both synthetic constructs and PCR products were blunt‐end cloned into pJET1.2 (ThermoFisher Scientific) according to manufacturer's instructions. DNA constructs were introduced into *P. gingivalis* through the natural competence method as described by Tribble et al., (2012), and successful transformants selected on erythromycin (10 μg ml^−1^) containing BA plates. Mutants were confirmed by PCR of extracted genomic DNA (Promega Wizard Genomic DNA), with PCR products sequenced at GATC Biotech to establish insertion of *ermF* at the expected position.

**Table 1:** Bacterial strains used in this study

*Porphyromonas gingivalis* strain	Relevant characteristic(s)	Source
ATCC 33277	Wild‐type, type strain	ATCC
∆*ompA1*	*ompA1* (PGN_0729) deletion mutant of ATCC 33277 (Em^R^)	This study
∆*ompA2*	*ompA2* (PGN_0728) deletion mutant of ATCC 33277 (Em^R^)	This study
∆*ompA1A2*	*ompA1* (PGN_0729) and *ompA2* (PGN_0728) deletion mutant of ATCC 33277 (Em^R^)	This study
∆*ompA2 *+* * pT‐COW‐A2	∆*ompA2* complemented mutant with *ompA* operon promoter and *ompA2* gene (from ATCC 33277) on pT‐COW plasmid (Tc^R^)	This study

Em^R^, erythromycin resistant; Tc^R^, tetracycline resistant.

### 2.3 Complementation of *∆ompA2*

A complementation construct for the *ompA2* gene was created by overlap extension PCR, fusing the *ompA2* gene to the 300 bp upstream flank of *ompA1* (primers listed in Table S2) and containing restriction sites for *Bam*HI and *Sal*I to allow cloning into pT‐COW plasmid (Gardner, Russell, Wilson, Wang, & Shoemaker, 1996). Clones were confirmed by sequencing and introduced into the ∆*ompA2* strain as described above. Clones containing the pT‐COW‐*ompA2* plasmid (or the empty pT‐COW plasmid) were selected on tetracycline (3 μg ml^−1^) agar.

### 2.4 Antibiotic protection assay to determine bacterial invasion of OK‐F6 monolayers

Antibiotic protection assays were carried out as previously described (Suwannakul et al., 2010). Briefly, OK‐F6 cells were seeded at 1 × 10^5^ cells/well in a 24‐well plate and cultured overnight for cells to adhere. The confluent cell monolayer was washed with PBS and nonspecific binding sites were blocked with 2% bovine serum albumin (BSA) in DKSFM at 37°C for 1 hr at 5% CO_2_. A cell count was made by trypsinizing one well to determine the multiplicity of infection (MOI). *P. gingivalis* was taken from a 3‐day old BA plate and adjusted to an MOI 1:100 in DKSFM and incubated with the OK‐F6 monolayer for 90 min at 37°C, 5% CO_2_. Following incubation, unattached extracellular bacteria were removed through PBS washes, and the total number of bacteria associated was determined by lysing epithelial cells in sterile dH_2_O. Lysates were diluted and plated on BA and incubated anaerobically for 7 days. Invasion by *P. gingivalis* was measured by incubating the infected monolayer with metronidazole (200 μg ml^−1^) to kill external adherent bacteria, and incubated for 1 hr at 37°C at 5% CO_2_. Cells were then washed thoroughly with PBS, lysed in dH_2_O, serially diluted, plated on BA and incubated anaerobically for 7 days. The number of viable bacteria was determined by seeding additional wells with *P. gingivalis* simultaneously with the rest of the experiment, and performing colony counts from serial dilutions on BA plates. CFUs were enumerated to determine the total number of bacteria associated with the cells (adherent and invaded) and the number of bacteria invaded, and expressed as a percentage of the viable count of the initial inoculum (Suwannakul et al., 2010).

To assess the influence of OmpA2 predicted surface peptides, standard antibiotic protection assays were carried out as before with the following alteration. After BSA incubation, an additional incubation step was included by incubating cells with 50 μg ml^−1^ of each peptide for 1 hr, followed by addition of bacteria in the presence of peptide (50 μg ml^−1^) for 90 min before processing as above. Biotinylated peptides were purchased from CovalAb (Cambridge, UK) or Isca Biochemicals Ltd., (Exeter, UK) in freeze‐dried format and resuspended in PBS and stored at −20°C before use.

### 2.5 Bacterial biofilm assay

*P. gingivalis* cells were seeded at an OD_600_ 0.05 into the wells of a 96‐well polystyrene plastic plate. After anaerobic incubation for 72 hr, total cell growth was measured at OD_600_ to ensure total growth was similar (within OD_600_ 0.1 of each strain), then planktonic cells were removed and the remaining biofilm layer washed with PBS and adherent cells stained with 1% Crystal Violet solution. Biofilms were assessed visually, using an inverted microscope (Nikon Eclipse TS100) at × 400 magnification connected to a digital camera. After thorough washing with PBS, biofilm formation was evaluated by measuring the OD_570_ following ethanol extraction of the Crystal Violet.

### 2.6 Fluorescence binding assay of extracellular peptide loops to OK‐F6 monolayers

Biotinylated peptides were bound to 1.0 μm yellow‐green NeutrAvidin^®^‐labeled FluoSpheres^®^ (ThermoFisher Scientific) at a concentration of 50 μg ml^−1^ and stored at 4°C in the dark. OK‐F6 cells were seeded at 1 × 10^5^ cells/well in a 96‐well polystyrene plate and incubated at 37°C, 5% CO_2_ overnight. After the cell monolayer was washed with PBS, 0.1% BSA in DKSFM was applied for 1 hr before cells were washed in PBS before peptide‐bound FluoSpheres^®^ were incubated with the cells at a concentration of 1:100 (cells:FluoSpheres^®^) for 4 hr at 37°C and 5% CO_2_. Fluorescence was measured at 488 _nm_/515 _nm_ (ex/em), using a TECAN Infinite 200 Pro before and after removal of non‐adherent FluoSpheres^®^ and data was corrected for any discrepancies in total FluoSpheres^®^ applied. BSA coated FluoSpheres^®^ and a scrambled version of peptide 4 were used as a control. For immunofluorescence imaging, cells were seeded onto coverslips in a 24‐well microtitre plate at the same seeding density, with peptide addition as above. After removal of peptides, the cells were fixed in 4% paraformaldehyde before thorough PBS washes. Cell membranes were stained, using WGA‐Texas Red^®^‐X Conjugated antibody (Invitrogen) according to the manufacturer's instructions. The coverslips were then mounted on glass slides, using ProLong^®^ Gold Antifade Mountant with DAPI (ThermoFisher Scientific) and imaged using an Axiovert 200 mol L^‐1^ Microscope (Zeiss).

### 2.7 Gingipain activity assay

Whole cell gingipain activity was determined, using overnight cultures of *P. gingivalis* pelleted and washed in PBS before the OD_600_ adjusted to 1.0. Bacteria (10 μl) were added to a 96‐well microtitre plate containing 1 μl 1 mol L^−1^ L‐cysteine, 100 μl TNCT buffer (50 mmol L^−1^ Tris‐HCl pH 7.5, 150 mmol L^−1^ NaCl, 5 mmol L^−1^ CaCl_2_, 0.05% Tween‐20) and incubated at room temperature for 10 min. For Arg‐gingipain activity, 100 μl of 0.4 mmol L^−1^ substrate *N*‐α‐Benzoyl‐L‐arginine *p*‐nitroanilide was added or 100 μl 0.4 mmol L^−1^ toluenesulfonyl‐glycyl‐L‐prolyl‐L‐lysine p‐nitroanilide for Lys‐gingipain activity and Abs_405 nm_ was measured to determine the rate of gingipain activity.

Secreted gingipain activity was measured as described by Chen, Nakayama, Belliveau, and Duncan (2001), using culture supernatants after cells were pelleted from an overnight culture adjusted to OD_600_ 1.0. Supernatants (50 μl) were added to a 96‐well MTP containing 100 μl PBS, 1 mmol L^−1^ L‐cysteine and either 200 μmol L^−1^ α*N*‐benzoyl‐L‐arginine‐7‐amido‐4‐methylcourmarin substrate (Arg‐gingipain) or 10 μmol L^−1^ *t*‐butyloxycarboyl‐Val‐Leu‐Lys‐7‐amido‐4‐methylcourmain substrate (Lys‐gingipain), and incubated at room temperature for 10 min before the reaction terminated, using 200 μmol L^−1^ *N‐*α‐tosyl‐L‐phenylalanine chloromethyl ketone (TPCK) (Arg‐gingipain) or 500 μmol L^−1^ *N*‐α‐p‐tosyl‐L‐lysine chloromethyl ketone (TLCK) (Lys‐gingipain). Released 7‐amido‐4‐methylcourmarin was measured at 365 _nm_/460 _nm_ (ex/em).

### 2.8 Outer membrane vesicle quantification

Liquid bacterial cultures were precleared by differential centrifugation. Bacterial cells were pelleted by centrifugation at 8000*g* for 10 min. Cell‐free supernatants were subject to further centrifuge steps (10,000*g* for 30 min) to remove cellular debris. Supernatants were diluted 1/10 in sterile PBS. Bacterial OMVs were analyzed by tunable resistive pulse sensing (TRPS), using a qNano instrument (iZON Science Ltd). Diluted samples (40 μl) were applied to the upper fluid cell above an NP100 nanopore stretched at 45.5 mm. A voltage (42 V) and positive pressure (2 mbar) was applied to cause unidirectional flow of OMVs through the nanopore. Samples were compared to CPC100B calibration particles of known size (114 nm) and concentration (1 × 10^13^ particles ml^−1^) and analyzed, using the iZON Control Suite software that was provided with the instrument. OMV concentration was normalized to the OD_600_ of the corresponding bacterial culture.

### 2.9 Statistics

All studies were carried out in a triplicate format in at least 3 independent experiments, with results expressed as the mean ± SEM. Statistical significance measured using students’ *t*‐test and One‐way ANOVA with the Greenhouse–Geisser correction (Graphpad Prism) after normality was assured, using the D'Agostino‐Pearson omnibus test. Statistical significant was assigned if *p *<* *.05.

## 3 Results

### 3.1 OmpA modulates *P. gingivalis* biofilm formation in vitro

In order to examine the function of OmpA and its two subunits in biofilm formation and host–pathogen interaction, we created isogenic mutants of the *ompA1*,* ompA2* , and the entire *ompA* operon (*ompA1A2*) in the same parent *P. gingivalis* ATCC 33277 strain (Naito et al., 2008). Single *ompA1* and *ompA2* and double *ompA1A2* knock‐out constructs were created and the DNA construct was introduced to wild‐type *P. gingivalis* through natural competence (Tribble et al., 2012). Mutants were confirmed by PCR and sequencing (data not shown). In addition, the presence and absence of OmpA proteins in the three strains was performed, using SDS‐PAGE, and using an anti‐OmpA antibody according to Nagano et al., (2005) to check for lack of polar effects of our OmpA1 mutant on OmpA2 expression, with no changes in OmpA2 expression observed in this strain (not shown). It should also be noted that we performed experiments on three separate original erythromycin resistant colonies (i.e. separate clones), to eliminate any potential influence of extraneous mutations. We also assessed the gross morphology of these strains, using TEM (Fig. S1), which demonstrated altered outer membrane morphology in a small number of the population (3–4%), as previously observed, but more strongly for the double than single mutants, again as has been observed by others (Iwami et al., 2007).

**Figure 1:**
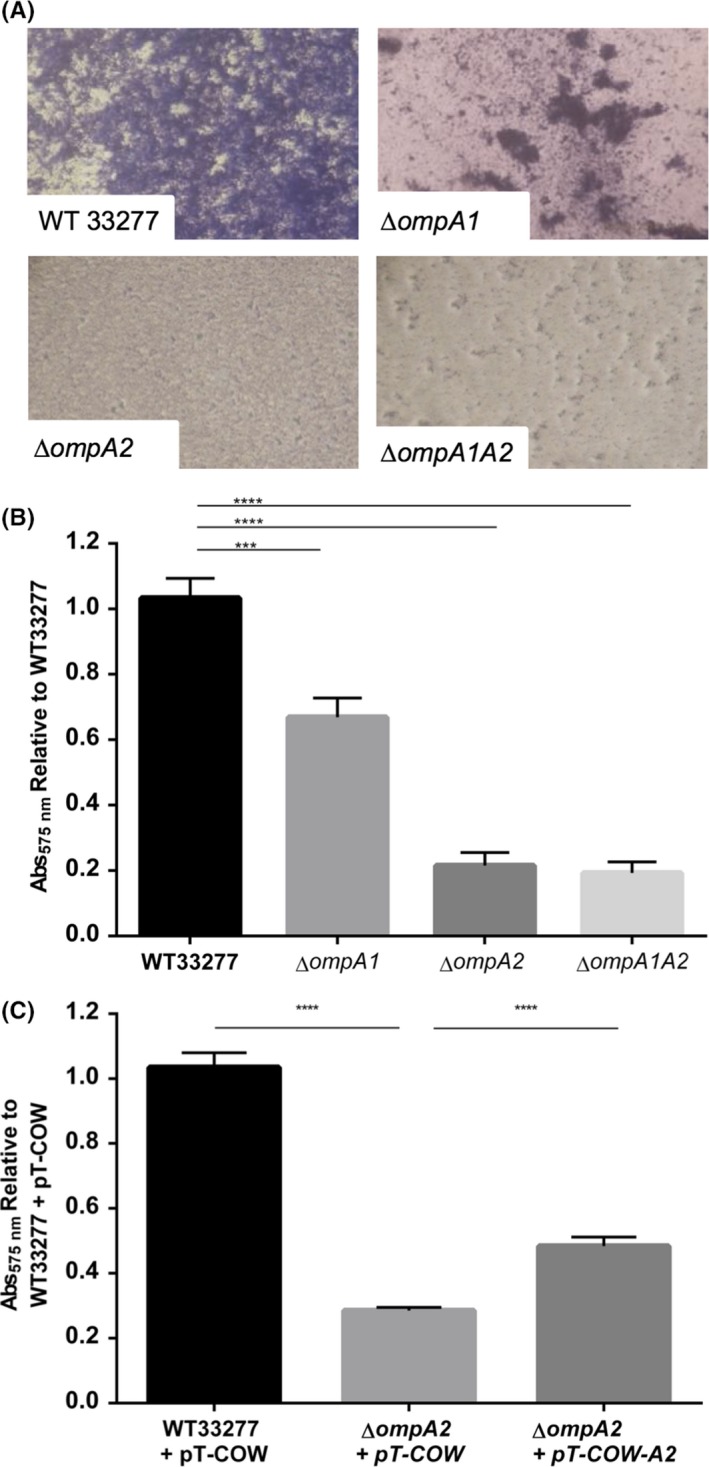
Biofilm formation in vitro. OD_600 nm_ 0.05 cultures were seeded and grown anaerobically for 72 hr, and biofilm stained with 1% Crystal Violet. Biofilms were imaged at 400× magnification (A), before Crystal Violet extracted and absorbance measured (OD_570_) to quantify biofilm formation (B). The ∆*ompA2* mutant was complemented and biofilm examined (C). Statistical significance was determined by students’ *t*‐test and designated as ***p <* *.001, ***^*^ *p *<* *.0001 (*n* = 3)

Biofilm formation is an important virulence factor for oral microbes as this is the basis of plaque formation in vivo*,* we therefore used a standard Crystal Violet assay to examine the ability of wild‐type and *ompA* mutant *P. gingivalis* strains to adhere to and form a biofilm on polystyrene microtitre plate surfaces. The overall growth (planktonic and biofilm) of the wild‐type and *ompA* mutants was observed through measuring the absorbance before removal of planktonic cells, with no difference in growth detected. We observed that biofilms derived from all three mutants were more fragile during washing and lifted easily from the plate bottom. Microscopic analysis showed that while the ∆*ompA1* strain is still capable of forming a biofilm in patches, the ∆*ompA2* and ∆*ompA1A2* mutants form very sparse biofilms (Fig. 1A). Quantification using Crystal Violet supported this observation with the ∆*ompA2* single and *∆ompA1A2* double mutant showing 4.5‐fold and 8.8‐fold reduction in biofilm formation, respectively (*p *< .05). Since the ∆*ompA2* mutant showed a phenotype similar to the ∆*ompA1A2* that was clearly different from the ∆*ompA1* mutant (only 40% reduction), the *ompA2* gene was complemented *in trans* using a plasmid containing the *ompA2* gene under the control of the *ompA* operon promoter. Reintroduction of the *ompA2* gene into the *ΔompA2* strain partially restored its ability (approx. twofold increase) to form a biofilm (*p *<* *.0001), but did not fully complement compared to wild‐type containing the empty pT‐COW plasmid for reasons we cannot explain.

As mentioned above, it is known that fimbriae play a role in biofilm and human cell interactions and it is possible that our mutants might have altered fimbrial properties. However, like previous studies (Iwami et al., 2007), we observed fimbrial‐like structures around our bacteria in thin‐section TEM (Fig. S1A) and also detected fimbrial protein in cell envelope preparations of our strains (Fig. S1C), indicating this is not likely to be the cause of observed phenotypes.

### 3.2 OmpA2 is involved in adhesion and invasion of oral epithelial cells

**Figure 2:**
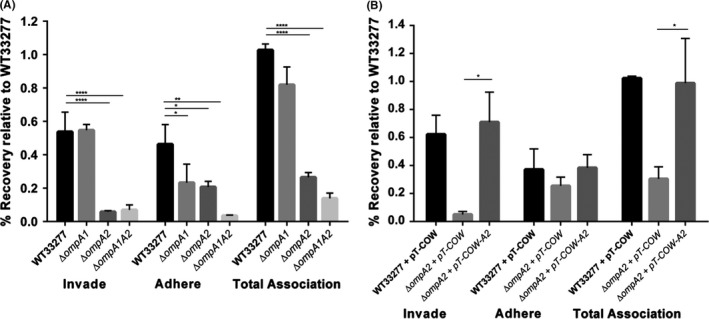
Bacterial adhesion and invasion of OK‐F6 monolayers by wild‐type, ∆*ompA1,* ∆*ompA2* and ∆*ompA1A2* mutants. *P. gingivalis* was incubated with a monolayer of OK‐F6 at a MOI 1:100 as described for invasion assays. Invasion was defined as the percentage of the inoculum protected from metronidazole killing. Total association was defined as the number of bacteria that have adhered to the OK‐F6 cell and invaded. Adherence was calculated from subtracting invasion CFUs from the total association. Each % value was determined by calculating the CFUs recovered as a percentage of the viability of that strain, and corrected to wild‐type *P. gingivalis* total association (=1). Wild‐type and mutant strains were evaluated for invasion and adherence efficiency (A), and the complemented *ompA2* mutant (B) assessed. Statistical significance was determined by students’ *t*‐test and designated as *p <* *.05, **p <* *.01, ***^*^ *p *<* *.0001 (*n* = 3). Error bars are ± SEM

Antibiotic protection assays were carried out with wild‐type *P. gingivalis* and the ∆*ompA* isogenic mutants to examine the role of OmpA in interactions with oral epithelial cells. Figure 2A shows differential adherence to OK‐F6 cells for all three mutants, with the double ∆*ompA1A2* mutant showing the least adherence. Compared to wild‐type bacteria, adherence by ∆*ompA* mutants was reduced 2.1‐fold, 2.45‐fold, and 13‐fold for the ∆*ompA1,* ∆*ompA2* and ∆*ompA1A2* mutants, respectively (*p *<* *.05 single mutants, *p *<* *.01 double mutant). The invasive capability of *P. gingivalis* was significantly (*p *<* *.0001) affected by the deletion of the ∆*ompA2* gene and the entire ∆*ompA1A2* operon, with a 10‐ and 8.3‐fold reduction in invasion, respectively; while in contrast, deletion of *ompA1* had no effect on invasion, but lead to a reduction in attachment and indicate that OmpA2 plays a more crucial role in cell interactions than OmpA1. Therefore, given its clearly stronger role in host–cell interaction, we therefore focus on OmpA2 in the remainder of this study, but acknowledge that OmpA1 may play a secondary, lesser role. As the deletion of *ompA2* demonstrated a reduction in invasion and adhesion of OK‐F6 cells, we again used our ∆*ompA2* (+ pT‐COW‐*ompA2*) complementation strain and assessed levels of invasion and adhesion, observing that both adherence and invasion were restored to wild‐type levels (Fig. 2B). These data again indicate that the OmpA2 protein has the largest influence on cell interactions in this system. No significant change was observed in the viability of the mutants in cell culture media in comparison to the wild‐type strain indicating that this phenotype was not due to reduced cell viability of the mutant strains (Fig. S2).

**Figure 3:**
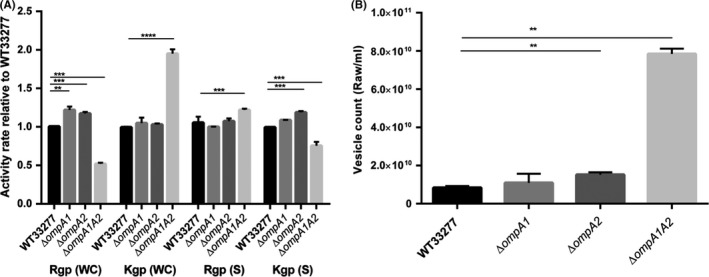
Gingipain activity and outer membrane vesicle production analysis of ATCC 33277 wild‐type and ∆*ompA* mutants. (A) Arg‐ and Lys‐gingipain activity assessed as previously described (Iwami et al., 2007). WC, whole cell, S = supernatant. (B) Vesicle number was quantified using a qNANO (iZON Science). Error bars are ± SEM (*n* = 3). Statistical significance was determined by students’ *t*‐test and designated as **p <* *.01, ***p <* *.001, ****p <* *.0001

In addition, and since gingipains are known to be major virulence factors for interaction of *P. gingivalis* with host cells, we assessed the activity of whole cell (WC) and secreted (S) fractions of wild‐type, Δ*ompA1* and Δ*ompA2* mutants alongside the double mutant using substrates specific for lysine (Kgp) and arginine (Rgp) gingipains. We observed no significant differences between cellular (WC) gingipain activity between Δ*ompA1* and Δ*ompA2* mutants with both being approximately 15% higher for Rgp, but not Kgp than wild‐type bacteria. In contrast, the *∆ompA1A2* double mutant displayed increased and decreased WC activity for Rgp and Kgp activity, respectively (Fig. 3A). When secreted activity (from culture supernatants) was assessed, there were again subtle differences (~18%) in activity of wild‐type compared to Δ*ompA2 ,*but we do not consider any of these large enough to explain the phenotypes observed for the Δ*ompA2* strains.

Other roles proposed for OmpA in previous studies included influences on outer membrane vesicle formation (Iwami et al., 2007). To assess this, we also quantified vesicle production, using a qNANO (iZON Science), which showed a slight increase (1.8‐fold) in vesicle formation for the ∆*ompA2* mutant, and a large increase in vesicle formation in ∆*ompA1A2* (Fig. 3B).

### 3.3 OmpA2 surface regions directly interact with oral epithelial cells

**Figure 4:**
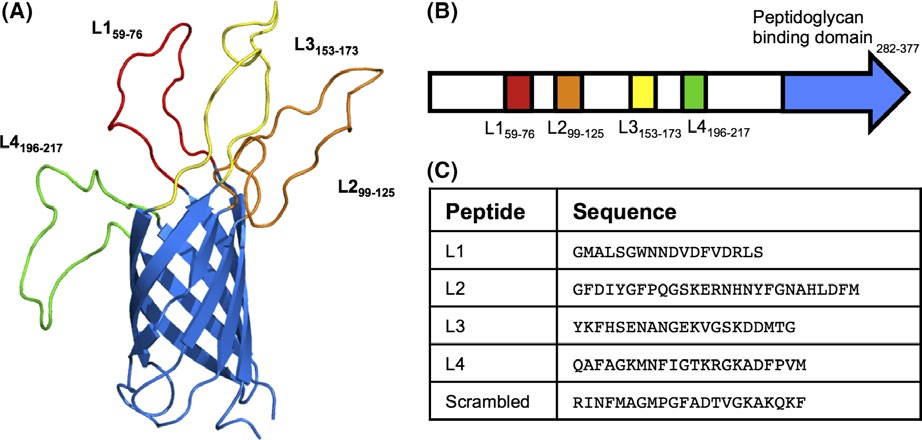
In silico analysis of OmpA2 protein and extracellular loops. (A) Structure modeling of OmpA2, displaying transmembrane β‐barrel and predicted extracellular loops, L1‐L4. N‐terminal α‐helix and C‐terminal peptidoglycan domain have been removed for display purposes. (B) Schematic representation of the location of the extracellular loops (colour corresponding to β‐barrel image) and predicted peptidoglycan‐binding domain (pale green) in the *ompA2* gene. Predicted extracellular loops sequences (C) were commercially ordered and Biotin‐tagged

**Figure 5:**
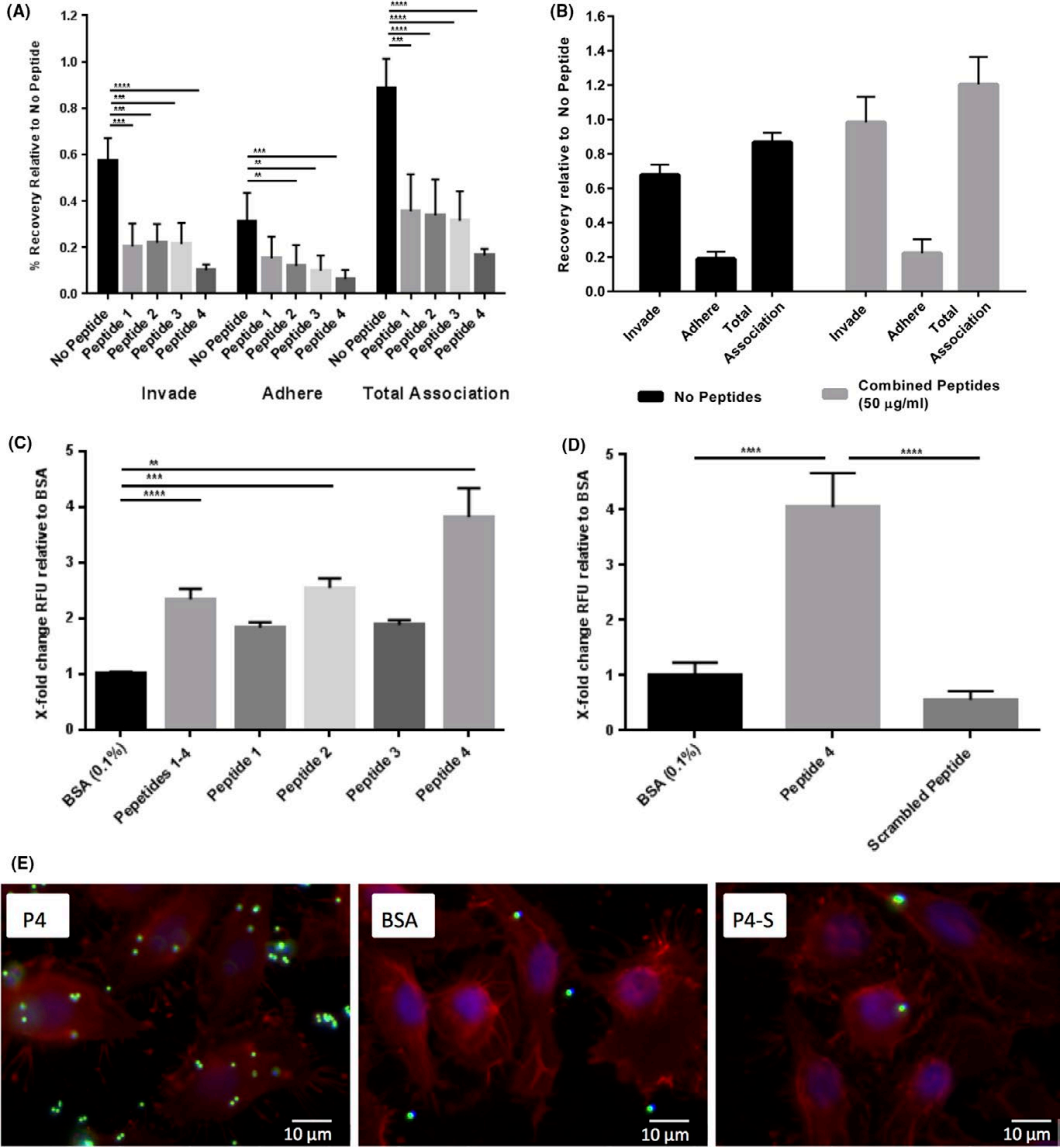
OmpA2 extracellular loops display direct binding to oral epithelial cells. Antibiotic protection assays were carried out with wild‐type *P. gingivalis* in the presence of each extracellular loop individually at 50 μg ml^−1^ (A), or at 50 μg ml^−1^ total concentration for all four loops (B). (C) Extracellular loop peptides were bound to NeutrAvidin^®^‐green fluorescent microspheres at 50 μg ml^−1^ and incubated with a monolayer of OK‐F6 cells and the total fluorescence at 488 _nm_/515 _nm_ (ex/em) recorded as a measure of the quantity of extracellular loop peptides bound to cells, relative to BSA‐coated microspheres. (D) A scrambled peptide was used as a control. (E) Immunofluorescence images of peptide 4‐bound microspheres (P4) incubated with OK‐F6 monolayers and imaged at ×100 magnification, BSA‐coated microspheres (BSA) and scrambled‐peptide‐bound microspheres (P4‐S). NeutrAvidin^®^‐green microspheres are visualised in the Green channel (488 nm) with WGA‐Texas Red^®^ (red, 549 nm) highlighting cell membranes and DAPI (blue) for cell nuclei. Statistical significance was determined by students’ *t*‐test and designated as **p <* *.01, ***p <* *.001. ***^*^ *p *<* *.0001. Error bars ± SEM. Scale bars are 10 μm. BSA, bovine serum albumin

We next investigated the molecular basis of the interaction between OmpA2 and human oral epithelial cells. It is well established that the OmpA protein displays structural similarities between different bacterial species, with a highly conserved integral outer membrane β‐barrel domain, whereas the extracellular loops are highly variable both in structure and size (Pautsch & Schulz, 2000; Schulz, 2002). In addition, these surface‐exposed extracellular loops have been shown to be involved in a variety of functions, acting as phage‐docking receptors in *E. coli* OmpA (Koebnik, 1999), or interaction with host cells, such as the OmpA‐like proteins found in *Neisseria gonorrhoeae* and *Coxiella bruneii* (Martinez, Cantet, Fava, Norville, & Bonazzi, 2014; Serino et al., 2007). To help further understand the role of the *P. gingivalis* OmpA protein in the interaction with host cells, the structure was studied in silico and modeled using online analysis software Phyre2 (http://www.sbg.bio.ic.ac.uk/phyre2/) and RaptorX (http://raptorx.uchicago.edu/) as well as beta‐barrel prediction programmes such as PRED‐TMBB (http://biophysics.biol.uoa.gr/PRED-TMBB/). Bioinformatic analysis by all three in silico methods predicted eight transmembrane beta sheets forming a beta barrel domain with four peptide loops located in this N‐terminal beta‐barrel domain (L1_59‐76_, L2_99‐125_, L3_153‐173_ and L4_196‐217_) predicted to be exposed at the cell surface, while the C‐terminal peptidoglycan‐associated domain (displaying structural homology to *E. coli* OmpA) was predicted to sit in the bacterial periplasm (Fig. 4 A and B). The orientation of the protein and location of surface exposed loops was supported by all software prediction programmes used. We surmised that these predicted exposed, extracellular peptide loops might be involved in the interaction with human oral epithelial cells. To test this prediction, biotin‐labeled peptide loops 1–4 were commercially synthesized, alongside a biotin‐tagged scrambled peptide version of Loop 4 (Fig. 4C) as a negative control. We then used these peptides alongside wild‐type *P. gingivalis* ATCC 33277 in adhesion and invasion blocking studies to establish which OmpA2 loops are important in mediating interactions with host cells. Peptides 1–4 significantly decreased *P. gingivalis* adherence (2.7–5.7‐fold) and invasion (2–4.9‐fold) when applied individually (at 50 μg ml^−1^) (Fig. 5A), with peptide 4 (QAFAGKMNFIGTKRGKADFPVM) having the greatest effect showing a 5‐fold reduction in adherence and invasion of wild‐type *P. gingivalis* (*p *<* *.001). However, if all four peptides were combined to a total concentration of 50 μg ml^−1^ (i.e. 12.5 μg ml^−1^ each peptide) no effect on adherence and invasion was observed (Fig. 5B), indicating a concentration dependent effect.

To further dissect the interaction between OmpA2 extracellular loops and oral epithelial cells we examined the ability of the peptides to mediate the interaction of inert latex beads with oral epithelial cells. Biotinylated peptides were linked to NeutrAvidin^®^‐coated fluorescent microspheres (FluoSpheres^®^) and applied to a monolayer of OK‐F6 cells. As before peptide 4 had the greatest effect in this assay, producing a 4‐fold increase in fluorescence intensity compared to BSA‐coated microsphere controls. Of the other peptides, only peptide 2 and the four peptides in combination (1/4 concentration of each) significantly (*p *<* *.001, and *p *<* *.0001 respectively) mediated interaction of the beads with OKF6 cells. To further confirm specificity we compared peptide 4‐mediated microsphere binding to that of a scrambled version of peptide 4 (RINFMAGMPGFADTVGKAKQKF). We observed that peptide 4 bound to cells 8‐fold greater than the scrambled peptide which, in turn, had similar adhesion levels to that of the BSA control (Fig. 5D and E). The fluorescent microspheres bound to the cells were enumerated from at least 3 images by counting the number of spheres bound per cell (visualised using DAPI‐stained nuclei and whole membranes, WGA‐TexasRed^®^) to quantify the level of binding in Figure 5E. Peptide 4‐bound microspheres (7.1 microspheres/cell) displayed an 8‐fold higher level of binding compared to BSA‐bound microspheres (0.88 microspheres/cell) and a 16‐fold higher level of binding compared to the scrambled peptide (0.41 microspheres/cell), all significant to *p *<* *.0001 using *t*‐test (data not shown). These data indicate that the presence of extracellular loop 4 of OmpA2 is sufficient for host–cell interaction of inert particles and suggest a direct interaction between peptide 4 and molecules on the surface of human oral epithelial cells.

## 4 Discussion

The major outer membrane protein (OmpA) is an integral protein in the surface of many Gram‐negative bacterial membranes and is predicted to be expressed by all Gram‐negative bacteria (Beher, Schnaitman, & Pugsley, 1980). OmpA has conserved N‐terminal β‐sheet forming residues indicating a strong selective pressure on the β‐barrel motif (Wang, 2002). Large sequence variations are observed in the extracellular loops (Pautsch & Schulz, 1998), implying a sequence specialised to their role and environmental niche. In this investigation, we have explored the role of *P. gingivalis* OmpA and its surface loops in the interaction with host cells and in a vertebrate systemic infection model.

Biofilm formation is an important virulence factor in many bacteria, but especially in oral microbes as the biofilm on tooth structures forms the basis of dental plaque (Cook, 1998). The OmpA protein of *E. coli* has been shown to be involved in biofilm formation through overexpression of *ompA* on a variety of hydrophobic surfaces (Ma & Wood, 2009; Orme et al., 2006). Due to the predicted structural similarity of *P. gingivalis* OmpA to *E*. *coli* OmpA, we investigated the role of OmpA in *P. gingivalis* biofilm formation. Our data demonstrate that the loss of the entire OmpA protein heterotrimer complex or even the OmpA2 subunit alone causes significant reduction in biofilm formation on inert surfaces, suggesting a specific role for the OmpA2 protein in the interaction with the environment surrounding *P. gingivalis*.

Previous studies of *P. gingivalis* biofilm formation have investigated the importance of gingipains for both single‐species biofilm and multi‐species biofilm formation with other periodontal pathogens such as *Treponema denticola* and *Tannerella forsythia* (Bao et al., 2014; Yamada, Ikegami, & Kuramitsu, 2005; Zhu et al., 2013). In addition, the major fimbriae of *P. gingivalis* are known to be important in biofilm formation (Kuboniwa et al., 2009; Yamamoto et al., 2011). However, we observed fimbrial like structures associated with our mutant strains and similar levels of cell‐associated and secreted Rgp and Kgp gingipain activity, indicating that our data appear to reveal a specific role for OmpA2 in biofilm formation.

*P. gingivalis* adherence and invasion of oral epithelial cells has previously been reported by several investigators (Chen et al., 2001; Njoroge et al., 1997) and *P. gingivalis* has been found to reside in the interior of buccal cells in vivo (Rudney & Chen, 2006; Rudney et al., 2005). Here we report for the first time the involvement of the OmpA protein in interactions with oral epithelial cells, the principal cell type with which *P. gingivalis* comes into contact in the oral cavity. In particular we highlight a specific and significant role for the OmpA2 subunit and its surface exposed loops in this interaction. Intriguingly our data reveal that while adherence is reduced in the ∆*ompA1* mutant strain in a similar fashion to the ∆*ompA2* strain, the number found intracellularly is similar to the wild‐type strain, indicating that it is the OmpA2 protein that is involved in interactions leading to internalization. This observation is in contrast to reports suggesting that the entire OmpA1A2 protein heterotrimer is necessary for binding to extracellular matrix molecules (Murakami, Hasegawa, & Nagano, 2014), however our data shows clear evidence for OmpA2 being the dominant subunit in epithelial cell interaction.

The importance of OmpA in mediating interactions of *P. gingivalis* with host cells has been observed previously in the context of endothelial cell adhesion where increased adherence of wild‐type *P*. *gingivalis* was observed on TNFα‐stimulated cells. However, no increase in ∆*ompA1A2* adherence was seen, and purified OmpA heterotrimer prevented the interaction of wild‐type *P. gingivalis* with endothelial cells in concentrations as low as 0.25 ng ml^−1^ (Komatsu et al., 2012). In addition, our previous studies examining gene expression of *P. gingivalis* in bistable ‘hyperinvasive’ sub‐populations of *P. gingivalis* indicated upregulation of OmpA in two strains tested (Suwannakul et al., 2010), further supporting our observations here. Furthermore, our data indicate that the interaction between OmpA and human epithelial cell proteins is likely to be direct given that synthetic peptides generated from predicted surface exposed loops of the OmpA protein specifically mediate the interaction of inert latex beads with human epithelial cells in vitro and exogenous addition of loop peptides to the media abrogated *P. gingivalis* invasion of epithelial cells. Our finding that isolated OmpA2‐derived peptides has an effect on cellular interactions of *P. gingivalis* also argues strongly against any pleiotropic effects of the OmpA mutations on fimbrial expression or gingipain activity.

Similarly, our data assessing OMV production by the *ompA* mutant strains are not suggestive of a role for OMV production in the invasive phenotype differences we observe, that is, because we see a reduction in invasion to the same extent between ∆*ompA2* and ∆*ompA1A2*, despite a large difference in vesicle number formation, we therefore posit that vesicle formation does not cause the decrease in invasion we show here. Equally, due to the similarities between Δ*ompA1* and Δ*ompA2* mutant phenotypes and the evidence we provide that synthetic peptide versions of OmpA2 peptide loops can both block host–cell interactions but also direct interaction of inert beads with human epithelial cells; we propose the reduced invasion phenotype of the ∆*ompA2* mutant is due to the lack of the OmpA2 protein subunits.

Although the involvement of surface exposed OmpA loops is a new finding in *P. gingivalis* research, it has been previously observed for a range of other important human pathogens. The extracellular loops of *E. coli* OmpA are essential for the invasion of human brain endothelial cells (Maruvada & Kim, 2011; Prasadarao et al., 1996), with mutations in loops 1 and 2 causing loss of pathogenicity (Mittal, Krishnan, Gonzalez‐Gomez, & Prasadarao, 2011). The human pathogen, *Coxiella burnetii*, known for causing Q fever, also displays extracellular loop specificity for host interaction, with deletion of loop 1 showing a significant reduction of bacterial internalization in lung epithelial cells (Martinez et al., 2014). In addition to human pathogens, elegant work by Weiss et al. has also shown a role for OmpA in bacterial–host interactions as part of the symbiotic relationship of the tsetse fly (*Glossina morsitans*) and the Gram‐negative bacterium, *Sodalis glossinidius,* whereby introduction of recombinant *E. coli* K12 OmpA resulted in a pathogenic phenotype for *Sodalis*. Weiss et al. also demonstrated comparisons of OmpA alignments in pathogenic *E. coli* and symbiotic *Sodalis* displaying significant insertions and substitutions in extracellular loop 1 which were not present in the pathogen‐associated form of OmpA (Weiss et al., 2008). Altogether, this evidence indicates that the role of OmpA extracellular loops in bacterial–environmental interactions (be that inert or cellular surfaces) may be a widespread mechanism of host cell interaction.

While our data indicate a direct interaction between OmpA extracellular loops and human epithelial cells we at present have no evidence what its receptor might be. In the case of endothelial cells data was provided that OmpA might interact via E‐selectin (Komatsu et al., 2012). However, we have no evidence that this is the case in epithelial cells where expression of E‐selectin is unclear given conflicting evidence of its presence or absence (Moughal, Adonogianaki, Thornhill, & Kinane, 1992; Pietrzak, Savage, Aldred, & Walsh, 1996). In the case of *E. coli* K1 meningitis strains evidence suggests a role for gp96, a cell surface glycoprotein related to heat shock proteins (Prasadarao et al., 1996) in OmpA‐mediated interactions with brain endothelial cells, and identifying extracellular loops 1 and 2 of the *E. coli* OmpA protein (which have low homology with the *P. gingivalis* respective loops) as being especially important in gp96 interaction (Mittal & Prasadarao, 2011; Mittal et al., 2011). The identity of the receptor in oral epithelial cells currently remains elusive, although in current work we are attempting to use the biotinylated peptides to probe for interacting partners from epithelial cells.

In conclusion, we have identified a role for the OmpA2 protein of *P. gingivalis* in the formation of biofilms, and adherence and invasion of oral epithelial host cells. In particular, we have shown the importance of the extracellular surface regions of OmpA2 in the interaction with host cells. Our data indicate a potential key role for these peptides in cellular interactions and thus suggests the exciting possibility of using surface protein‐derived peptide loops as potential anti‐adhesive therapeutics or immunization antigens (as has been used for other *P. gingivalis* proteins (Cai, Kurita‐Ochiai, Kobayashi, Hashizume, & Yamamoto, 2013)) but also OmpA as a potential drug target for treatment of periodontal disease via targeting the keystone pathogen, *P. gingivalis*.

## Funding Information

Kathryn Naylor was funded by a The University of Sheffield Faculty of Medicine, Dentistry and Health studentship, Mary Connolly was supported by the Harry Bottom Trust and Magdalena Widziolek by 2012/04/A/NZ1/00051 grant from National Science Center (NCN, Krakow, Poland). Matthew Hicks was funded by a BBSRC grant (BB/JO16322/1) to Dr. Graham Stafford.

## Conflict of Interest

None declared.
