# Supplementary figures and images for: Role of OmpA2 surface regions of Porphyromonas gingivalis in host–pathogen interactions with oral epithelial cells
Source: Microbiologyopen. 2016 Sep 6;6(1):e00401. doi: 10.1002/mbo3.401 (PMC5300881; doi:10.1002/mbo3.401)

# Supplementary Fig 1

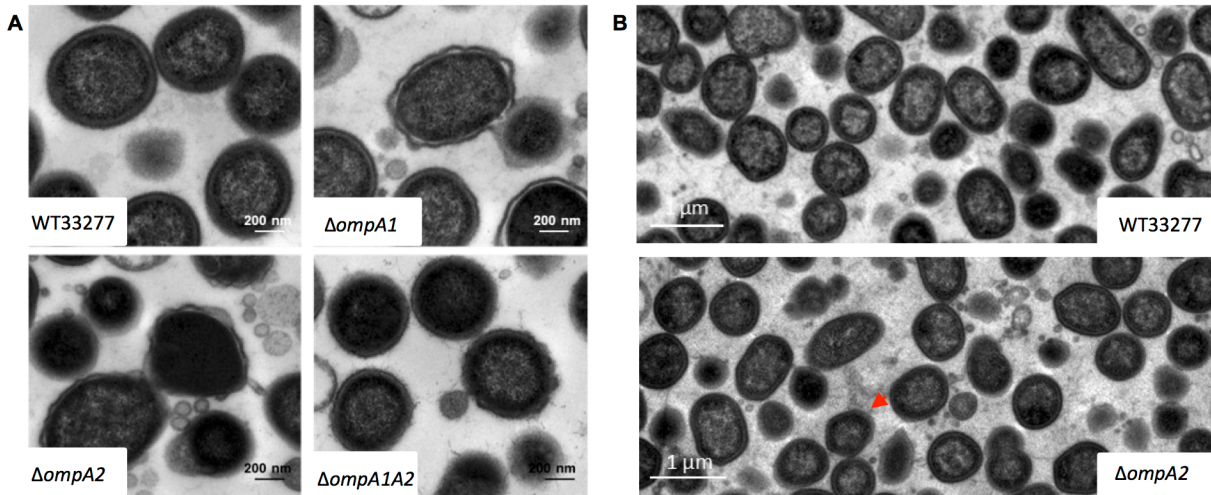

Supplement: Supplementary file 1 [file MBO3-6-0-s001.pdf]

Fig S2

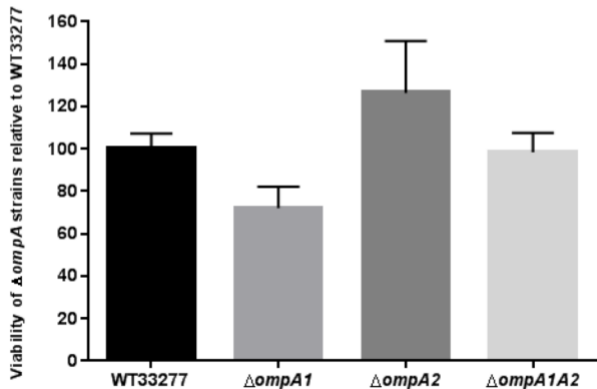

Supplement: Supplementary file 2 [file MBO3-6-0-s002.pdf]
