# Supplementary material for: Role of OmpA2 surface regions of Porphyromonas gingivalis in host–pathogen interactions with oral epithelial cells
Source: Microbiologyopen. 2016 Sep 6;6(1):e00401. doi: 10.1002/mbo3.401 (PMC5300881; doi:10.1002/mbo3.401)
Supplement: Supplementary file 3 [file MBO3-6-0-s003.docx]

**Supplementary Tables**

| **Primer Name** | **Sequence (5’ – 3’)** |
| --- | --- |
| *ompA1* F1 For | TGGAAGTTAGCATCGCCAAC |
| ***ermF* For** - *ompA1* F1 Rev | **CGGGCAATTTCTTTTTTGTCAT**GCCTATTCGCTACACAAAT |
| *ompA1* F1 Rev - ***ermF* For** | CATTTGTGTAGCGAATAGGC**ATGACAAAAAAGAAATTGCCCG** |
| *ompA1* F2 For - ***ermF* Rev** | CCCGCAAGTGCTAATAATA**CGAAGGATGAAATTTTTCAGGGAC** |
| ***ermF* Rev** - *ompA1* F2 For | **GTCCCTGAAAAATTTCATCCTTCG**TATTATTAGCACTTGCGGG |
| *ompA1* F2 Rev | GATCAAGGTAGTAGGTTAC |
| *ompA2* F1 For | ATAGGTTCTCTTTCTGTCGG |
| ***ermF* For** - *ompA2* F1 Rev | **CGGGCAATTTCTTTTTTGTCAT**CTGTATGTCATTTTATATTATCC |
| *ompA2* F1 Rev - ***ermF* For** | GGATAATATAAAATGACATACAG**ATGACAAAAAAGAAATTGCCCG** |
| *ompA2* F2 For - ***ermF* Rev** | CTCGTCATGAGACAGCCTCAGC**CGAAGGATGAAATTTTTCAGGGAC** |
| ***ermF* Rev** - *ompA2* F2 For | **GTCCCTGAAAAATTTCATCCTTCG**GCTGAGGCTGTCTCATGACGAG |
| *ompA2* F2 Rev | TTCACTTTGTCTTTCAGATCCTCT |

| **Primer Name** | **Sequence (5’-3’)** |
| --- | --- |
| *ompA1* Promoter For | CCGGAGAATCTTCTTTGCAGC |
| *ompA1* Promoter Rev (*ompA2* For) | GCTAATAATAAAGATTTAGCTTTCAT(AGTTTTACTTTTCTAAGTG) |
| (*ompA1* Promoter Rev) *ompA2* For | (CACTTAGAAAAGTAAAACT)ATGAAAGCTAAATCTTTATTATTAGC |
| *ompA2* Rev | TTATTCCGCTGCAGTCATTACTAC |

**Supplementary Figures**

**Table S1. Primer list for *ompA1* and *ompA2* gene knock outs. Bold indicates erythromycin (*ermF*) resistance gene sequence.** F1 and F2 indicate the flanking region before (F1) and after (F2) the gene of interest.

**Table S2. Primer list for *ompA2* gene complementation.** Parenthesis indicate overlap.

**Fig. S1. Outer membrane analysis of wild-type *P. gingivalis* ATCC 33277 and ∆*ompA1*, ∆*ompA2*, and ∆*ompA1A2* mutants. TEM micrographs of whole cell *P. gingivalis* wild-type and ∆*ompA* mutants including fimbrial detection.** (A) Liquid cultures (OD_600nm_ 1.0) were fixed in glutaraldehyde and dehydrated with ethanol before embedding in avaldite. Grids were stained using uranyl acetate and lead citrate as previously described (Reynolds 1963). A) demonstrates membrane disruption in all *ompA* mutants, however this is only observed in a small fraction of the population, approx.. 3-4%,, as shown in B) by a lower magnification image. Examples of fimbriae-like electron densities are indicated by red arrows in all images. **(C) detection of major fimbrial protein (FimA) in outer membrane fractions using anti-FimA antibody (1:3000) for wild-type and ∆*ompA* mutants as indicated.**

**Fig. S2. Bacterial viability in *P. gingivalis* wild-type and *ompA* mutants.** Viability was calculated from CFU counts from standard antibiotic protection assays. No significant differences were observed. Error bars are ± SEM (n=3).
